# Comparative transcriptome profiling of potato cultivars infected by late blight pathogen *Phytophthora infestans*: Diversity of quantitative and qualitative responses

**DOI:** 10.1016/j.ygeno.2023.110678

**Published:** 2023-09

**Authors:** C.A. Agho, E. Kaurilind, T. Tähtjärv, E. Runno-Paurson, Ü. Niinemets

**Affiliations:** a Chair of Crop Science and Plant Biology, Estonian University of Life Sciences, Kreutzwaldi 1, Tartu 51006, Estonia; b Centre of Estonian Rural Research and Knowledge, J. Aamisepa 1, 48309 Jõgeva, Estonia; c Estonian Academy of Sciences, Kohtu 6, Tallinn 10130, Estonia

**Keywords:** Potato, Late blight resistance, RNAseq, Transcriptome, Differentially expressed genes

## Abstract

The Estonia potato cultivar Ando has shown elevated field resistance to *Phytophthora infestans*, even after being widely grown for over 40 years. A comprehensive transcriptional analysis was performed using RNA-seq from plant leaf tissues to gain insight into the mechanisms activated for the defense after infection. Pathogen infection in Ando resulted in about 5927 differentially expressed genes (DEGs) compared to 1161 DEGs in the susceptible cultivar Arielle. The expression levels of genes related to plant disease resistance such as serine/threonine kinase activity, signal transduction, plant-pathogen interaction, endocytosis, autophagy, mitogen-activated protein kinase (MAPK), and others were significantly enriched in the upregulated DEGs in Ando, whereas in the susceptible cultivar, only the pathway related to phenylpropanoid biosynthesis was enriched in the upregulated DEGs. However, in response to infection, photosynthesis was deregulated in Ando. Multi-signaling pathways of the salicylic-jasmonic-ethylene biosynthesis pathway were also activated in response to *Phytophthora infestans* infection.

## Highlights

• Potato cultivar Ando has maintained a long-lasting high-field resistance to *Phytophthora infestans.*
• A comparative transcriptome analysis was performed to gain insights into its resistance mechanism.
• Compared to a susceptible cultivar, about a five-fold greater number of differentially expressed genes were found in the highly resistant cultivar Ando.
• Several disease-resistant pathways were significantly enriched in Ando and photosynthesis was deregulated.
• Multi-signaling pathways of the salicylic-jasmonic-ethylene biosynthesis pathway were also activated in response to *P. infestans* infection at 72 h post-inoculation.

## 1 Introduction

Potato (*Solanum tuberosum* L.) is globally the third most important food crop, after rice and wheat, and it is cultivated in all continents except Antarctica [17]. Potato production has increased dramatically, especially in developing countries, with a worldwide increase of 21% in the past two decades, indicating its growing importance as a staple food source [17]. However, the productivity of potato is hampered by several biotic stresses among which late blight caused by the oomycete pathogen *Phytophthora infestans* (*P. infestans*) is the most dreaded one [108]. Despite significant efforts in its treatment, potato late blight, a re-emerging potato disease [42], has remained the primary limitation on potato production globally with an estimated annual loss of about €6.1 billion [2]. Under favorable conditions, *P. infestans* can easily spread from plant to plant and can destroy the entire field [41]. *P. infestans* is the most important potato disease in Europe [53], and first appeared there >160 years ago [106]. The pathogen can also infect other solanaceous plants, such as tomato [41]. Due to its rapid plant-to-plant spread and potential to quickly decimate the entire potato field, *P. infestans* poses a severe threat to Estonian potato production [106]. Breeding for drought tolerance was the major concern in potato breeding program in Estonia, until the first outbreak of late blight disease in early 1972 in Jõgeva potato breeding fields, which changes the focus to resistance breeding [108].

Though investment in conventional agriculture has been beneficial in yield improvement, it is not sustainable in the long term [96]. Management of late blight disease mostly depends on the repeated application of fungicides, which can lead to a slow erosion of disease control due to a gradual loss of sensitivity of the targeted pathogen population to the fungicide and leaves production systems vulnerable to the development of highly resistant strains of a pathogen to these chemicals or traits over time [116]. In addition, it also increases production costs and environmental risks. There is a continuous chase for resistant genes in potato as genetic resistance to late blight is often transient due to breakdown by the rapidly evolving pathogen. The current knowledge in this area is still not robust enough [143]. Investigations on more potato cultivars are still necessary to collect information on general resistant genes and response patterns. The use of late blight resistant potato varieties is still considered an important aspect in the control of this disease [141]. The method requires no action from potato growers and its use poses no harm to the environment [72]. Besides, this approach is usually cheaper and compatible with other disease management techniques [72]. However, this traditional breeding approach for effective late blight disease management can be challenging and requires tremendous genetic resources and efforts, especially in sourcing new resistance genes. As a result of continuous breeding efforts, the Centre of Estonian Rural Research and Knowledge has developed and released a late blight resistance cultivar Ando [107,122], also suitable for organic cultivation [122,128]. For >40 years, the cultivar Ando has maintained a durable resistance to *P. infestans,* despite the dynamic plant-pathogen-environmental interactions in nature [109], and the highly diverse and changing *P. infestans* populations in the Baltic region and nearby [67,68,104]. On the other hand, the Dutch (breeding company Agrico) potato cultivar Arielle is very susceptible (S) to *P. infestans* [110]. However, the molecular mechanisms underlying the resistance of Ando to late blight infection are still not known. It is essential to understand its resistance mechanism at the molecular level, like examining the differential expression in response to the pathogen at the gene level that would allow for the identification of candidate genes linked to resistance to *P. infestans* for use in breeding to develop resistant cultivars.

The cultivar Ando has a round tuber with shallow tuber eye depth, tastes good, and has a medium to high starch content (https://www.europotato.org/varieties/view/Ando-E). One of cv. Ando's parents, the Latvian cultivar Agra, is considered a cultivar of medium to low resistance, while the other parent, line 382–48, has a well-known source of resistance, *Solanum demissum* in its pedigree [109]. *P. infestans* resistance genes R1 (Rpi-R1) and R2 (Rpi-R2) from *S. demissum* regularly were used previously for cross-breeding in potato breeding programs in Estonia [61,108]. Currently, the Rpi-R1 gene is identified with SSR markers in most of the moderately and highly late blight-resistant cultivars including ‘Ando’ [61,108]. The cultivar Ando has been assessed for late blight resistance since its breeding at

the Centre of Estonian Rural Research and Knowledge in 1966 and it was included in the Estonian Variety List in 1977 [109]. Its foliar resistance to late blight has been assessed as medium to high [European Cultivated Potato Database; https://www.europotato.org/varieties/view/Ando-E; [122]).

The recent development of high-throughput whole transcriptome shotgun sequencing, known as RNA-seq, has made it possible to study genotypic response to biotic and abiotic stresses in plants, and comprehensively dissect the molecular mechanism involved [38,40,54]. With the release of plant genome sequences including the potato (Potato Genome Sequencing Consortium, 2011), RNA-seq has become a powerful tool for studying transcriptomic differences in potato cultivars in response to early and late blight infection [38,52,54,125,143], and provides a novel approach to study the resistance at the molecular scale, by monitoring thousands of genes simultaneously. The use of RNA-seq technique to look at differentially expressed genes (DEGs) in *P. infestans* infected resistant and susceptible potato genotypes allowed identification of 40 effector targets [3]. Likewise, genes encoding transcription factors and protein kinases, and four NBS-LRR proteins were among the 3354 DEGs identified in late blight resistant potato genotype SD20 [143]. The findings from RNA-seq analysis were used to improve the late blight interaction model in resistant potato genotype JAM1–4 [149].

In the present study, we used RNA-seq to investigate the changes in gene expression underlying the resistance mechanisms induced by *P. infestans* in the highly late blight resistant cultivar Ando in comparison to the susceptible cultivar Arielle. Moreover, this is the first report underlying the qualitative and quantitative transcriptomic profile in highly late blight resistant cultivar Ando in response to *P. infestans* infection, compared to a susceptible cultivar. To our knowledge, this is also the first report showing the molecular mechanism underlying this resistance in Ando based on RNA-seq analysis. Ando and Arielle are of two different genetic backgrounds. They were infected with a highly diverse *P. infestans* strain, collected from a large-scale conventional field and characterized by Runno-Paurson et al. [106] and in unpublished research by [155]. The time points: before and 72 h after inoculation by *P. infestans,* were used for potato leaves from the two cultivars, which were then subjected to RNA sequencing (RNA-seq) analysis. Late blight pathogen exhibits a biphasic infection pattern, including the biotrophic phase (early infection stage), where minimal symptoms are exhibited by the plant, and a necrotrophic phase where leaf necrosis and pathogen sporulation are clearly visible to the naked eye [16,47,143,153]. The mechanism involved in defense responses at both phases (PAMP-triggered immunity (PTI) in the biotrophic phase and effector-triggered immunity (ETI) during the necrotrophic phase) often overlap and have a similar set of downstream defense responses [31,120], such that important insight into the overall defense response can be achieved by studying either of the two phases [102,116] or both [38,52,54,143]. However, the mechanism activated in the later stage (necrotrophic phase) is often stronger, has a longer duration, and is robust against pathogen infection [31,44,63]. The start of the necrotrophic phase of the late blight pathogen is mainly activated 72 h after the infection [3,16]. Indeed, analysis of differential gene expression at 72 hpi might be more informative as no significant changes in gene induction were found in moderately resistant and susceptible potato cultivars during an early stage of the *P. infestans* infection [103]. In our study, the post-inoculation stage of 72 h was chosen to allow for sufficient time of infection and elicit detectable expressional changes in both the resistant and susceptible cultivars.

## 2 Methods

### 2.1 Plant and late blight material and late blight inoculation

Two widely cultivated potato cultivars, Ando (resistant, R) and Arielle (susceptible, S) maintained at the Centre of Estonian Rural Research and Knowledge (METK) in Jõgeva, Estonia, were selected for the study. The cultivars were grown in a growth chamber under the long-day condition set at 20 °C with a 16 h/8 h (light/dark) cycle and 70% relative humidity [67]. *P. infestans* isolate Ve5–13 of pathotype 1.3.4.7.10.11 [106], the dominating race in Europe and Nordic countries [1,51,55,70,75,105] was used. The pathogen was collected from a potato field (large-scale conventional field type) in Veriora village, Põlva County, Estonia (58.0059° N, 27.3496° E). The isolate was maintained on rye —B medium in a petri dish at 17 °C in the dark. Freshly produced sporangia were harvested from plates by physical scraping into sterile water. The resulting inoculum was adjusted to 4 × 10^4^ sporangia/ml under a microscope before being cooled down at 4^o^ C for 2 h to promote the release of motile zoospores [126] before inoculation.

Fully-expanded leaves from six-week-old potato plants of both cultivars were inoculated with prepared inoculum by spraying onto the leaves until runoff, and mock inoculation (referred to as before inoculation, or healthy plant throughout the manuscript) was done by spraying with the same volume of sterilized water for each cultivar and the experiment was repeated three times. Plants were kept in a growth chamber set at 18 °C with a 16 h/8 h (light/dark) cycle and 90% relative humidity [38]. For RNA extraction, leaf samples from treated and untreated plants were collected at 72 hpi and immediately shock-frozen in liquid nitrogen and kept at −80 °C before RNA extraction [143]. All three biological replicated samples were used for RNA extraction.

### 2.2 RNA extraction and sample preparation

Total RNA was isolated from the frozen plant material using Norgen Total RNA Isolation Kit (Norgen Biotek Corp.) according to the manufacturer's instructions. A NanoDrop 2000 spectrophotometer (Thermo Fisher) was used to detect RNA concentration and purity. For each sample, at least 20 μg of total RNAs was sent to Novogene Corp. (United Kingdom) for Illumina sequencing.

### 2.3 RNA sequencing, alignment, and data analysis

For *P. infestans* strain Ve5 treatment and control leaves for each cultivar, RNA sequencing libraries of all the 12 samples were constructed and sequenced by Illumina NovaSeq 6000 platform for 2 × 150 cycles at Novogene. Foliage RNA-seq raw data in BAM format have been submitted to the NCBI Sequence Read Archive (SRA) under the accession number PRJNA759282 (https://www.ncbi.nlm.nih.gov/bioproject/PRJNA759282). Raw data (raw reads) of FASTQ format was filtered with fastp software [24]. The potato genome sequence (SolTub_3.0, GCA_000226075.1) was acquired from the NCBI Genomes database, and the corresponding genome annotation (https://www.ncbi.nlm.nih.gov/genome/?term=Solanum+tuberosum) [125]*.* The trimmed pair-end reads of each sample were aligned to the reference genome using HISAT2 v2.1.0 [69]. Raw counts were generated by counting the total number of reads that were mapped to the gene region. R (version 4.1.1) package DESeq2 [80] was used to normalize expression levels and perform differential expression analysis assuming a negative binomial distribution [6]. Here the obtained read counts are normalized using regularized log transformation function of DESeq2 in the R package to adjust for differences in library size or sequencing depth, library composition, assuming negative binomial distribution by applying scaling factors (median ratio normalization) [6,76]. Normalized RNA-seq read counts of the samples were examined using principal component analysis (PCA) and Pearson's correlation heatmap as quality control to check for grouping and reproducibility [76]. Differential expression analysis between before and after inoculation of samples was performed using DESeq2 [80]. Raw *P* values were adjusted for multiple testing using a false discovery rate [12]. Genes with an adjusted *P* < 0.05 found by DESeq2 were assigned as differentially expressed (DEG). Log2 Fold Change (FC) > 0 was considered upregulated, whereas log2 FC < 0 was downregulated along with an adjusted *p*-value <0.05 for statistically significant results [20,32,98,127].

The data were divided for comparison into four groups: cultivar Ando, 72 hpi (Ando_D3); Ando, before inoculation (Ando_D0); Arielle 72 hpi (Arielle_D3), and Arielle before inoculation (Arielle_D0), and the following comparisons were made: Ando_D3 vs Ando_D0, Ando_D0 vs Arielle_D0, Arielle_D3 vs Arielle_D0, and Ando_D3 vs Arielle_D3. To visualize DEGs, the pheatmap R package [71] was applied to generate the heatmap and volcano plots based on the results of ggplot2 [135]. Differential expression of genes was deemed significant at adjusted *P* (*P*_adj_) < 0.05. To compare gene expression levels under different conditions, the distribution and dispersion of gene expression levels and FPKM among different samples were displayed by box plots. Gene ontology (GO) enrichment analysis was performed to get an overview of the functional category of the genes that participated in the *P. infestans* infection response [143]. The DEGs were classified into three main categories of biological process, cellular component, and molecular function. To compare and summarize the biosynthetic pathways that are activated in response to *P. infestans* infection the DEGs were mapped to the KEGG (Kyoto Encyclopedia of Genes and Genomes) database for the enrichment of pathways [143]. Statistical enrichment of DEGs in the GO and KEGG pathway was tested using the clusterProfiler of the R package (version 4.1.1) [146]. Adjusted *P* was estimated by the Benjamini-Hochberg FDR method [12], and differential enrichment of functional groups of genes was considered significant at *P*_adj_ < 0.05 [38]. Several authors consider RNA-seq methods and data analysis approaches to be robust enough such that independent validation by qPCR and/or other approaches is not deemed necessary [28,86,99,114]. In this study, experimental steps and data analyses were carried out according to the state-of-the-art procedures, and the focus of the study was on modifications of the suites of DEGs rather than on absolute expression levels of single DEGs that can be more precisely characterized by qPCR. Thus, we did not use qPCR analyses in the current study.

## 3 Results

### 3.1 *P. infestans* infection

**Fig. 1:**
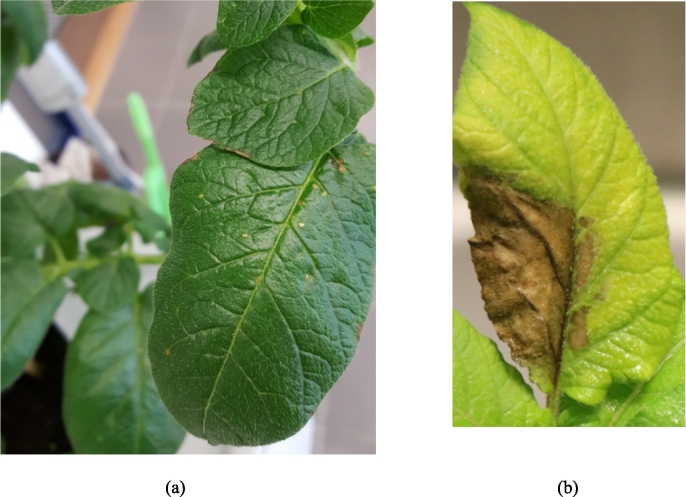
(a) Hypersensitive response (HR) on the leaves of Ando cultivar resistant to *P. infestans* and (b) Disease symptoms on Arielle susceptible cultivar with expanding sporulating lesion in response to *P. infestans*.

Ando is highly resistant to *P. infestans*, whereas Arielle is very susceptible. Leaves from six-week-old potato plants were inoculated with the *P. infestans* zoospore suspension and symptoms were observed over time. Localized HR-like lesion was observed on the leaves of Ando leading to pathogen growth arrest and thus no disease whereas water-soaked lesions were observed on the leaves of Arielle that resulted in severe disease development and visible pathogen growth and sporulation on the leaf surface (Fig. 1).

### 3.2 Alignment of RNA-seq reads and pattern of gene expression between Ando and Arielle in response to *P. infestans* infections

A total of 12 samples were used to produce RNA-seq reads using Illumina RNA-seq deep sequencing. Among all sequencing samples, approximately 173.6 Gb clean reads (with an average of 14.5 Gb clean bases) and 578,296,497 read pairs (obtained reads) were obtained (Table S1) and each sample had similar clean data and GC percentage (range from 42.26 to 43.13%). Between 65.35 and 89.98 million clean reads filtered from the raw reads of each library were aligned against the potato reference genome (Table S2). On average, 83.02% of the total reads were mapped to the reference genome, and 80.20% of reads of each sample were mapped to only one location of the reference genome with most of the reads mapping to exon regions and only a small proportion mapping to introns or intergenic regions (Table S2). Squared Pearson correlation coefficients (*r*^2^) calculated using all gene expression levels among untreated and *P. infestans* treated samples of the susceptible and resistant cultivar varied from 0.74 to 1.0 (Fig. S1), confirming the reliability and repeatability of the experiment in estimating the differential gene expression. The box plots of the values of the relative log2 fragments per kilobase of exon per million mapped fragments (log2FPKM) for each RNA-seq library displayed few distributional differences among the libraries (Fig. S1), suggesting similar transcription profiles.

**Fig. 2:**
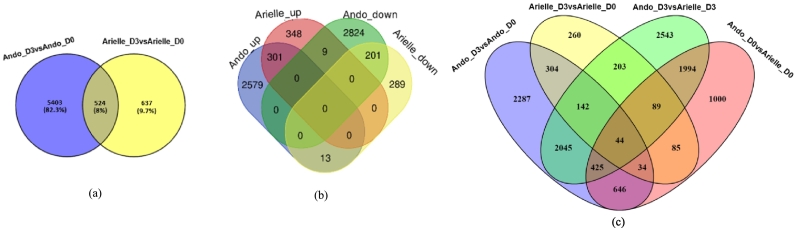
Venn diagram of differentially expressed genes (DEGs) between the resistant and susceptible potato cultivars in response to *P. infestans* infections. (a) DEGs distribution between Arielle at D3 vs Arielle at D0 and Ando at D3 vs Ando at D0 sets. (b) Venn diagram of common and specific significantly up-and down-regulated genes. (c) DEGs distribution among Arielle at D3 vs Arielle at D0, Ando at D3 vs Ando at D0, Ando at D3 vs Arielle at D3, and Ando at D0 vs Arielle at D0 sets. D0 and D3 are before, and 72 h after infection (72 hpi). up: upregulated; down: downregulated.

In the resistant cultivar Ando (incompatible interaction), 5927 DEGs were observed between Ando before (D0) and 72 h after infection, 72 hpi (D3) (Fig. 2a), where 2893 genes were upregulated compared to the healthy and 3034 genes were downregulated compared to the healthy (Fig. 2b). In the susceptible cultivar Arielle (compatible interaction), 1161 DEGs were observed between before (D0) and 72 hpi (D3). Among these DEGs, expression levels of 658 genes were upregulated compared to the healthy, and 503 genes were downregulated compared to the healthy (Fig. 2b). A total of 6564 genes were differentially expressed in the two genotypes considered together. Arielle_D3 vs. Arielle_D0 and Ando_D3 vs. Ando_D0 contained 524 DEGs that were common (Fig. 2a), and group-specific DEGs in Ando_D3 vs. Ando_D0 and Arielle_D3 vs. Arielle_D0 were 5403 and 637, respectively. A complete list and details of genes are provided (Table S3). When comparing resistant and susceptible cultivars under healthy conditions (D0), Ando_D0 vs. Arielle_D0 had 4317 DEGs, while at 72 hpi, Ando_D3 vs. Arielle_D3 had 7485 DEGs (Fig. 2c).

To have a quick overview of top genes differentially expressed in the compatible and incompatible interaction, the top 5% of DEGs based on log_2_FC were compared (Table S4). The expression levels of both differentially expressed upregulated and downregulated genes compared to the healthy in incompatible interaction (Ando) were higher than that of Arielle (compatible interaction). Among the top DEGs in Ando were nitrate transporter, glutaredoxin family genes, terpenoids biosynthesis genes such as sesquiterpene synthase, as well as flavonol synthase, 1-aminocyclopropane-1-carboxylate oxidase, MYC2, peroxidase, cytochrome P450, iron transporter protein, which were highly upregulated (>5 log_2_ FC), while in Arielle, peroxidases, transcription factor bHLH96 were among the top upregulated DEGs.

The genes differentially expressed in response to *P. infestans* were further analysed to identify specific as well as common upregulated and downregulated genes compared to the healthy in both compatible (Arielle) and incompatible (Ando) interactions. As shown in the Venn diagram (Fig. 2b), 301 upregulated DEGs, and 201 downregulated DEGs were common in both cultivars. 2592 genes were specifically upregulated in Ando, while in Arielle, 357 genes were specifically upregulated. Likewise, 2833 genes were specifically downregulated in Ando, while in Arielle, 302 genes were specifically downregulated. The expression levels of both specifically differentially expressed upregulated and downregulated genes in incompatible interaction (Ando) were higher than that of compatible interaction (Arielle), considering the top 5% of DEGs based on log_2_FC (Table S4). There were more specific upregulated and downregulated genes in the incompatible interaction, compared to compatible interaction. The common upregulated and downregulated genes are listed (Table S5), as well as the specifically upregulated and downregulated genes (Table S6).

Nine genes were identified as upregulated in Arielle but downregulated in Ando. Thirteen genes were upregulated in Ando but downregulated in Arielle (Table S7). Among these inversely regulated genes are a *stachyose synthase*, which was upregulated by about 5-fold in Ando, and downregulated by about 2-fold in Arielle, and *SlTCP3* and *Aspartyl-tRNA synthetase* genes which were downregulated in Ando, while in Arielle, they were upregulated.

### 3.3 Differential expression of genes linked to SA, JA, and ET signaling pathways

Among the 6564 annotated DEGs, we also found a group of regulatory genes and marker genes associated with the salicylic acid (SA), jasmonic acid (JA), and ethylene (ET) signaling pathways (Table 1). In the SA signaling pathway, phenylalanine ammonia-lyase (PAL) is a key enzyme, while *PR-1*, *PR-2* (*glucan endo-1, 3- β-glucosidase*), *NPR1*, *NDR1* (non-race-specific disease resistance 1) are marker genes in the pathways. Here, we identified 4 *PAL* genes in Ando, none in Arielle; 14 *PR-2* genes in Ando, 1 in Arielle; 1 *NDR1* in Ando, 1 *NPR1* in Arielle. All *PAL* genes in Ando were upregulated compared to the healthy and among the *PR-2* genes, seven were upregulated compared to the healthy. *NDR1* gene was only differentially upregulated in Ando compared to the healthy. In Arielle, the *PR-2* and *NPR1* gene were both upregulated compared to the healthy. In the JA signaling pathway, jasmonate ZIM-domain protein enzyme, LOX (lipoxygenase), AOS (allene oxide synthase), *MYC2*, and *PR-3* (chitinase) are key enzymes, and the genes encoding these enzymes are considered as the marker genes of the JA signaling pathway. We identified 3 *LOX* genes (two downregulated, one upregulated compared to the healthy), three downregulated *PR-3* genes*,* one upregulated *PR-3* gene*,* two *MYC2* genes that were both upregulated compared to the healthy and one upregulated *Jasmonate ZIM-domain protein* 1 gene in Ando. In Arielle, the *jasmonate ZIM-domain protein 3* gene was upregulated compared to the healthy. Marker genes for ET signaling aminocyclopropane-1-carboxylic acid synthase (ACC synthase) was upregulated in Ando compared to the healthy, as well as aminocyclopropane-1-carboxylic acid oxidase (ACC oxidase), where two were found upregulated in Ando, and three upregulated in Arielle (Table 1). *EIN3*-like transcription factors (*EIL*) were also identified. Here five *EIL/EIN3* were upregulated in Ando, while one *EIN3* was upregulated in Arielle*.* Similarly, Osmotin *OSML15* with about 4-fold upregulation was identified in Ando (Table 1).

**Table 1:** The key enzymes and marker genes of salicylate (SA), jasmonate (JA), and ethylene (ET) signaling pathways in *S. tuberosum* cultivars Ando and Arielle in response to *P. infestans* infection.

Gene ID	Desciption	Log2FC	Cultivar
PGSC0003DMG401021564	Phenylalanine ammonia-lyase, PAL	2.55	Ando
PGSC0003DMG400019386	Phenylalanine ammonia-lyase, PAL	0.62	Ando
PGSC0003DMG400031457	Phenylalanine ammonia-lyase, PAL	2.03	Ando
PGSC0003DMG402021564	Phenylalanine ammonia-lyase, PAL	1.88	Ando
PGSC0003DMG400025621	NDR1	1.20	Ando
PGSC0003DMG400005138	Glucan endo-1,3-*β*-glucosidase	0.77	Ando
PGSC0003DMG400024642	Glucan endo-1,3-*β-*glucosidase	−1.60	Ando
PGSC0003DMG400005021	Glucan endo-1,3-*β*-glucosidase	−1.81	Ando
PGSC0003DMG400026359	Glucan endo-1,3-*β*-glucosidase	3.21	Ando
PGSC0003DMG400000354	Glucan endo-1,3-*β*-glucosidase	−2.16	Ando
PGSC0003DMG400029476	Glucan endo-1,3-*β*-glucosidase	−1.28	Ando
PGSC0003DMG400016108	Glucan endo-1,3-*β*-glucosidase	−0.51	Ando
PGSC0003DMG400012702	Glucan endo-1,3-*β*-glucosidase	1.95	Ando
PGSC0003DMG400029830	Glucan endo-1,3-*β*-glucosidase	−1.21	Ando
PGSC0003DMG400016216	*β*-1,3-glucanase	−1.42	Ando
PGSC0003DMG400008727	*β*-1,3-glucanase	1.23	Ando
PGSC0003DMG400008368	*β*-1,3-glucanase	2.87	Ando
PGSC0003DMG401012862	*β*-1,3-glucanase	3.05	Ando
PGSC0003DMG402012862	*β*-1,3-glucanase	3.40	Ando
PGSC0003DMG400002930	Jasmonate ZIM-domain protein 1	2.96	Ando
PGSC0003DMG400032155	Lipoxygenase	−1.47	Ando
PGSC0003DMG400022894	Lipoxygenase	1.25	Ando
PGSC0003DMG400032207	Lipoxygenase	−1.94	Ando
PGSC0003DMG400000214	Chitinase	−1.09	Ando
PGSC0003DMG400021910	Chitinase	−0.80	Ando
PGSC0003DMG400025063	Class IV chitinase	0.87	Ando
PGSC0003DMG400019883	Chitinase	−2.34	Ando
PGSC0003DMG400012237	MYC2	5.68	Ando
PGSC0003DMG400007010	Myc2 bHLH protein	1.03	Ando
PGSC0003DMG400003058	Osmotin OSML15	4.01	Ando
PGSC0003DMG400005915	EIL1	0.96	Ando
PGSC0003DMG400029908	EIL1	0.43	Ando
PGSC0003DMG400027507	EIL3	1.59	Ando
PGSC0003DMG400002914	EIN3-binding F-box protein 1	1.86	Ando
PGSC0003DMG400015853	EIN3-binding F-box protein 1	1.12	Ando
PGSC0003DMG400004898	Transcription factor JERF1	1.49	Ando
PGSC0003DMG400040260	Glucan endo-1,3-*β*-glucosidase, basic isoform 1	2.96	Arielle
PGSC0003DMG400008160	BOP/NPR1/NIM1-like regulatory protein	2.32	Arielle
PGSC0003DMG400032119	Jasmonate ZIM-domain protein 3	1.50	Arielle
PGSC0003DMG400021426	1-aminocyclopropane-1-carboxylate synthase 3	2.81	Ando
PGSC0003DMG400017245	1-aminocyclopropane-1-carboxylate oxidase	−2.44	Ando
PGSC0003DMG400009720	1-aminocyclopropane-1-carboxylate oxidase	5.83	Ando
PGSC0003DMG400017247	1-aminocyclopropane-1-carboxylate oxidase	−2.33	Ando
PGSC0003DMG400017190	1-aminocyclopropane-1-carboxylate oxidase	1.68	Ando
PGSC0003DMG401021981	1-aminocyclopropane-1-carboxylate oxidase	−3.06	Ando
PGSC0003DMG400017190	1-aminocyclopropane-1-carboxylate oxidase	1.86	Arielle
PGSC0003DMG400017246	1-aminocyclopropane-1-carboxylate oxidase	1.28	Arielle
PGSC0003DMG400021476	1-aminocyclopropane-1-carboxylate oxidase	0.95	Arielle
PGSC0003DMG400030928	EIN3-binding F-box protein 1	0.98	Arielle

### 3.4 Differential expression of pathogenesis-related protein (PR) genes in response to *P. infestans*

In the incompatible interaction, PR-genes such as chitinases (PR-3), glucan endo-1, 3- β-glucosidase/1,3-beta glucanase (PR-2), protease/proteinase inhibitors (PR6), peroxidases (PR-9), ribonucleases (PR-10), thaumatin (PR-5), endoproteinase (PR-7) were among the upregulated genes (Table S8). In the compatible interaction, glucan endo-1, 3- β-glucosidase (PR-2), proteinase inhibitor (PR-6), and peroxidases (PR-9) were among the upregulated genes.

Apart from the PRs, twenty-four NBS-LRR resistance genes, seventeen upregulated and seven downregulated compared to the healthy were identified in Ando in this study (Table S8). In Arielle, two upregulated, and two downregulated NBS-LRR resistance genes were identified.

### 3.5 GO enrichment analysis

**Fig. 3:**
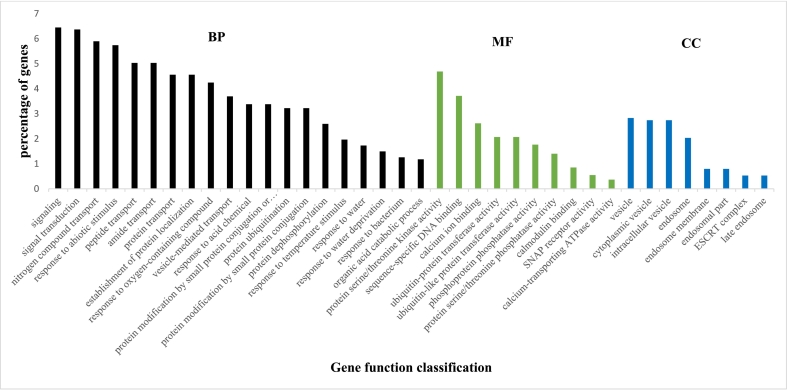
Gene ontology (GO) classification of upregulated DEGs in Ando_D3 vs Ando_D0. X-axis, three major functional categories of top 20 GO terms: biological process, molecular function, and cellular component; Y-axis, terms with percentages of DEGs in the major category. The percentage of genes is the ratio of the DEGs number to the background number in a certain process expressed in percentage. D0 and D3 are before and 72 h post-inoculation (72 hpi), respectively.

**Fig. 4:**
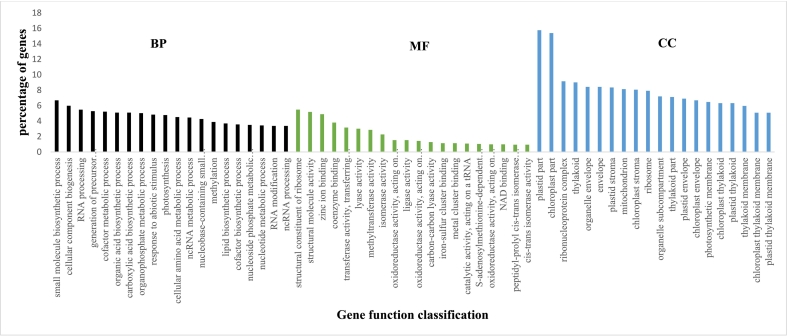
Gene ontology (GO) classification of downregulated DEGs in Ando_D3 vs Ando_D0. X-axis, three major functional categories of top 20 GO terms: biological process, molecular function, and cellular component; Y-axis, terms with percentages of DEGs in the major category. The percentage of genes is the ratio of the DEG number to the background number in a certain process expressed in percentage. D0 and D3 are before and 72 h post-inoculation (72 hpi), respectively.

In the resistance cultivar Ando, a total of 50 GO terms were significantly enriched in different biological processes, molecular function, and cell component categories among the upregulated DEGs between D0 and D3, in which biological processes GO domain was found to be highest (Table S9). In biological processes, 32 GO terms were significantly enriched, whereas, in the molecular function categories, 10 GO terms were significantly enriched, and in the cellular component category, 8 GO terms were significantly enriched. The top 20 GO terms of upregulated DEGs are shown (Fig. 3). Signaling, signal transduction, response to stimulus, transport processes were among the top 20 GO terms in the biological process category, while in the molecular function category, some of the top enriched terms include protein serine/threonine kinase activity, sequence-specific DNA binding, calmodulin, and calcium-binding, etc. In the cellular component category, significantly enriched terms were mainly located in the endosome and vesicles. In the susceptible cultivar, only 3 GO terms were significantly enriched in the upregulated DEGs, and these terms were in the molecular function category, and include oxidoreductase activity, iron ion binding, and monooxygenase activity (Table S9). Among the downregulated DEGs between D0 and D3 in the resistant cultivar, 211 GO terms were significantly enriched (Table S9), in which biological processes GO domain was found to be highest with 146 GO terms. This is followed by the cellular component category with 36 GO terms and the molecular function category with 29 GO terms. Small molecule biosynthetic process, RNA processing, carboxylic acid biosynthetic process, photosynthesis, etc., were among the top enriched terms in the biological processes in the resistant cultivar. Other significantly enriched terms include plastid organization, chloroplast organization, etc. The top 20 GO terms of downregulated DEGs are shown (Fig. 4). In the molecular function category, structural constituent of ribosome, structural molecule activity, lyase activity were among the top enriched terms. The majority of enriched terms in the cellular component category were related to photosynthetic machinery. In the susceptible cultivar, no GO terms were significantly enriched in the downregulated DEGs. The result indicates that the cultivars differ in their GO terms in response to infection, and there was a significantly higher number of GO terms observed exclusively in the resistant cultivar compared to the susceptible cultivar. Thus Ando (resistant cultivar) initiated a battery of response mechanisms to *P. infestans* as compared to Arielle (susceptible cultivar). Overall, in response to late blight infection in the resistant cultivars, GO terms related to pathogenesis responses such as signal transduction, protein serine/threonine kinase activity, and response to stimulus are upregulated, as well as mobilization of biological synthesis of various biological macromolecules and transport, while terms related to photosynthesis functions are downregulated.

### 3.6 Encyclopedia of genes and genomes (KEGG) pathway enrichment analysis of DEGs

**Fig. 5:**
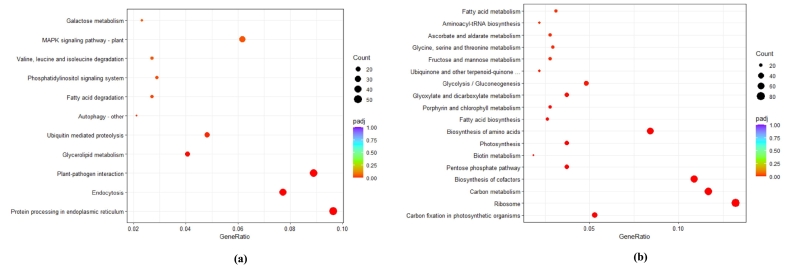
KEGG pathways assignment to differentially expressed genes (DEGs) in the resistant cultivar Ando for upregulated (a) and downregulated (b) differentially expressed genes compared to the healthy. Dot size and colour refer to the number of genes and the corrected *P*-value, respectively. The gene ratio is the ratio of the number of DEGs annotated in a given pathway term to the number of all genes annotated in the pathway term. A higher gene ratio indicates greater intensity. The padj value is the corrected *P-*value and ranges from 0 to 1, and a lower value indicates greater intensity. Only significantly enriched pathways are shown.

KEGG pathway enrichment analysis was performed to better elucidate the functions of the DEGs. For the resistance cultivar Ando, 11 KEGG pathways were significantly enriched in the upregulated DEGs (Fig. 5a). Annotated genes were found to be associated with KEGG pathways like protein processing in endoplasmic reticulum, plant-pathogen interaction, mitogen-activated protein kinase (MAPK) signaling pathway-plant, autophagy, as well as glycerolipid metabolism, fatty acid degradation, phospholipid signaling system, etc. 18 KEGG pathways were significantly enriched for downregulated DEGs in Ando, which include photosynthesis, fatty acid biosynthesis, fatty acid metabolism, carbon metabolism, etc. (Fig. 5b). In the susceptible cultivar Arielle, only 1 KEGG pathway phenylpropanoid biosynthesis was significantly enriched in the upregulated DEGs (Table S10), while in the downregulated DEGs, 3 KEGG pathways including starch and sucrose metabolism; cutin, suberin, and wax biosynthesis, as well as fructose and mannose metabolism, were significantly enriched (Table S10). A complete list and details of the KEGG pathways enriched in Ando and Arielle are provided (Table S10).

## 4 Discussion

### 4.1 RNA sequencing and gene expression profiling in susceptible and resistant potato cultivars in response to *P. infestans*

After sequencing, we obtained an average of 14.5 Gb clean reads for each sample. These clean reads were processed by differential expression analysis, GO analysis, and KEGG pathway enrichment analysis. We identified 5927 DEGs in Ando and 1161 genes in Arielle. A total of 2893 genes were upregulated in Ando compared to the healthy, and 3034 genes were downregulated compared to the healthy. A higher number of downregulated genes at 72 hpi have also been reported in resistant potato genotype SD20 in response to late blight infection [143]. We found 503 DEGs downregulated, and 658 DEGs upregulated in the susceptible cultivar (Arielle), compared to the healthy. These genes are important to study the functions affected by *P. infestans* infection in Arielle. Compared to the healthy, there were more upregulated and downregulated DEGs in Ando than in Arielle, indicating that more *P. infestans* resistance genes were expressed in the resistant cultivar after infection. The upregulated genes during pathogen and host interactions will help to gain insight into the mechanisms activated during defense [79,102,116,123]. This information will also serve as a valuable addition to the publicly available potato genomic information.

The expression profile in terms of DEGs of Ando relative to Arielle (Ando_D0 vs Arielle_D0) before infection was very different compared to after infection (Ando_D3 relative to Arielle_D3), with about double the amount of DEGs genes observed after infection. Thus, the transcriptional changes become increasingly stronger after infection. Likewise, changes in gene expression in response to infection were largely different for both the incompatible and compatible interactions compared to healthy, revealing that distinct defense responses could be initiated in response to the pathogen. Upregulated differentially expressed genes in the resistant cultivar were enriched in the biological processes related to external stimuli such as response to water, chemicals, temperature, oxygen-containing compounds (abiotic stressors), and biotic stressors. Thus, external stimuli, including *P. infestans* elicitor-like secretion can trigger a defense response in the host and result in differential expression [8]. The enriched GO term response to oxygen-containing compounds may account for function including reduction of oxidative stress [25,30,150]. The majority of annotated genes in incompatible interaction between soybean lines and *Phytophthora sojae* were related to response to biotic and abiotic stress [79].

### 4.2 Downregulation of photosynthesis-related genes in resistant cultivar

Significant enrichment of metabolic pathways related to photosynthesis was observed in the downregulated DEGs in the incompatible interaction. Biotic stress due to pathogens has been shown to strongly diminish the expression of genes related to photosynthesis, often leading to damage to photosynthetic machinery, and affecting the photosynthetic capacity of the leaves [14,19,21,38,46,49,152]. Thus, the resistant cultivar may have incurred a cost associated with defense response in the form of significant demand on resources that could have been channeled to photosynthesis. In this case, the plant places a higher priority on building a robust defense response in preference to growth [120]. This noticeable shift in priority from photosynthesis to defense response has been reported [27,34,52,94,121,137,139,149].

### 4.3 Pathways related to salicylic-jasmonic-ethylene acid signaling in response to *P. infestans* infection

Multiple strategies are deployed by plants for defense against biotic stressors. These include induced responses by signaling pathways regulated by salicylic acid (SA), jasmonic acid (JA), and ethylene signaling (ET) [124], whose activation promotes the production of localized and systemic defenses [78]. Defense against biotic stress is mainly regulated by SA-signaling, whereas JA and ET signaling are deployed against necrotrophs [30,129]. The start of the necrotrophic phase of the late blight pathogen is mainly activated 72 h after infection [3,16]. Cross-talk between SA and JA signaling plays a key role in defense against pathogens [38,89,119]. Activation of the SA-JA-ET signaling pathway has been shown to play a major role in late blight disease resistance [38,143]. This includes the activation of PAMP-induced defense response as shown in potato [50]. SA induction can trigger the activation of pathogenesis-related protein (PR) genes for PTI and ETI [132]. Marker genes for SA-induced defense response include *Phenylalanine ammonia-lyase* (*PAL*), *NDR1* (Non-race specific disease resistance 1), *PR-1*, *PR-2* (*glucan endo-1,3- β-glucosidase*) [143]. *PAL* is homologous to the salicylic acid (SA) marker gene *AtPAL1* in *Arabidopsis thaliana* [84]. Four *PAL* genes in Ando, none in Arielle, fourteen *PR-2* genes in Ando, and one in Arielle were identified, where all the *PAL* genes in Ando were significantly upregulated compared to healthy, and among the *PR-2* genes, seven were upregulated in Ando compared to healthy. In the susceptible cultivar Arielle, the *PR-2* gene was significantly upregulated. One *NDR1* was identified in Ando and was significantly upregulated, and none in Arielle. The role of SA in defense against *P. infestans* infection seems likely in this study. The previous study has shown the involvement of SA in the induction of cell death in resistance of mature *Nicotiana benthamiana* against *P. infestans,* and the absence of SA signaling makes the young plant susceptible [115]. The defense against *S. sclerotiorum* in *B. napus* was mediated by SA signaling [92]. Jasmonate ZIM-domain protein enzyme, *LOX* (lipoxygenase), *AOS* (allene oxide synthase), and *PR-3* (chitinase) are important genes involved in JA signaling [136,143]. *Jasmonate ZIM-domain protein 1* was upregulated in both Ando and Arielle, as well as some LOX genes and *PR-3* genes that were also upregulated in Ando. This suggests cross-talk between SA and JA in defense against necrotrophs, as suggested in previous studies in defense of potato against the necrotrophic bacterial pathogen *Dickeya solani* in both leaves and tuber [22]. Similarly, both SA and JA were induced in maize roots and leaves infected with *Colletotrichum graminicola* during the necrotrophic phase of infection [9]. Reports by previous studies have indicated the non-involvement of JA in susceptibility or partial resistance of potato against *P. infestans* infection [45,134]. However, JA induction played a role in defense against *P. infestans* in tomato [29,124] and potato [29]. In the necrotrophic pathogen *A. solani, it is argued that JA and not SA* signaling is responsible for the defense response [20]. The suppression of the JA defense pathway genes has been observed in potato during a compatible interaction with *P. infestans* [101]. However, JA induction enhances the resistance of tomato to *P. infestans* [124]. ACC synthase and ACC oxidase are major marker genes in the ET biosynthetic pathway [57,73,142,144]. In this study, ACC synthase was upregulated in the compatible interaction, whereas ACC oxidase, was found upregulated in both compatible and incompatible interaction. Furthermore, ETHYLENE INSENSITIVE 3 (*EIN3*) and *EIN3*-like transcription factors (*EIL*) are key regulators in the ET pathway [15,23,35,37,87,117,142,144]. In this study, the potato homolog of *EIL/EIN3* was upregulated in both compatible and incompatible interactions. *StOsmotin2* annotated as Osmotin *OSML15* in potato has been involved in ET response [38,136]. Osmotin *OSML15* was upregulated in incompatible interaction in this study. Our result suggests a role of ET signaling in defense response against *P. infestans* in potato at the necrotrophic stage, as also outlined in other reports [143]. ET signaling also plays a role in defense response against *Phytophthora sojae* in soybean [79]. Though ET/JA signaling is generally considered to be involved in resistance to infection by necrotrophic pathogens, whereas SA signaling is essential to prevent infection by biotrophic pathogens [44,85], a cross-talk among the SA-JA-ET signaling pathway is likely in this study [143], and underline the complexity of defense response against pathogens. The involvement of these pathways could also be genotype-dependent rather than correlating with resistance level [111]. Hence, more studies are needed to confirm the roles of these signaling pathways in defense response. Lastly, δ-8 sphingolipid desaturase was upregulated in incompatible interaction, as well as in the compatible interaction, suggesting the role of sphingolipid in plant defense response [13,38].

### 4.4 Transcription factors and pathogenesis-related proteins (PRs) in defense against *P. infestans*

Plant defense responses are controlled by several transcription factors (TFs), which bind to specific DNA sequences in their promoter regions to modulate the transcription of downstream resistance-related genes [113]. Many transcription factors, such as APETALA2/ethylene-responsive factor (AP2/ERF), basic helix-loop-helix (bHLH), MYB, WRKY, NAM/ATAF/CUC (NAC), basic-domain leucine-zipper (bZIP), ZF have been implicated in plant defense response ([5,43,54,66,83,100,112,143,145]; bZIP [7,43,66,83,95,112]. This study identified several transcription factors including AP2/ERF, WRKY, BHLH, ZF, MYB, bZIP, and NAC/NAM that were upregulated in both the resistant and susceptible cultivars.

The apoplast serves as the interaction interface between the pathogen and host during a pathogen attack, where successful defense leads to recognition and lysis of the pathogen [4]. Some of the apoplastic proteins are induced after a pathogen attack and are termed PR proteins [4]. Pathogenesis-related proteins (PRs) are a group of proteins that accumulate in plants after attack by a pathogen and are known to have antipathogenic activity against many phytopathogenic organisms such as *P. infestans*, *P. parasitica*, and *Fusarium* spp. [3,59,65,83,90,93,143]. These include β-1,3-glucanase (PR2), chitinases (PR3, 4, 8, and 11), thaumatin-like (PR5), protease inhibitor (PR6), endoproteinase (PR7), peroxidases (PR9), ribonucleases (PR10), plant defensins (PR12), thionins (PR13), lipid-transfer protein (PR14), etc. in various plants [3,83,130]. Many of the PRs were upregulated in this study, underlining their crucial role in plant defenses against pathogens.

### 4.5 DEGs associated with plant-pathogen interaction (PPI) pathway during *P. infestans* infection

The recognition and resistance response of plants to late blight is an intricate dynamic process involving two multifaceted stages: PTI and ETI [139,143,149]. PTI results from the recognition of pathogen-associated molecular patterns (PAMPs) at the initial stage of infection which is triggered by plant pattern recognition receptors (PRRs) [10,143,149]. The signal is delivered to the cell by endocytosis, which activates several protein kinases, such as the mitogen-activated protein kinase cascade, which is an important downstream part of PTI [10]. At a later stage of infection, when the pathogen gains entrance into the host, there is a recognition of pathogen virulence proteins (effectors) by resistance genes that result in ETI [36,139,143,149]. ETI and PTI exhibit similar defense responses that include calcium-mediated signaling, activation of mitogen-activated protein kinases (MAPKs), production of reactive oxygen species (ROS), transcriptional reprogramming, and biosynthesis of antimicrobial compounds, even though they have different recognition mechanisms [31]. There is an overlap between these two pathways during defense response resulting in local as well as systemic acquired resistance [39,139], and ETI have been regarded as an amplified PTI [31], with a longer duration and higher magnitude [120]. However, as the basal defense system becomes suppressed during a pathogen attack via the secretion of effectors by the pathogen that successfully attacks signaling components that regulate the basal defense system, the host becomes susceptible [60]. The KEGG pathways plant-pathogen interaction, endocytosis, autophagy, and mitogen-activated protein kinases (MAPK) signaling were among the significantly enriched terms in the upregulated DEGs in the resistant cultivar and may have contributed to the defense response against *P. infestans* infection. Studies have shown that mitogen-activated protein kinases (MPKs)-mediated photosynthetic inhibition plays an active role during ETI [120]. Members of the MPK genes have been reported to coordinate the growth-defense trade-off in plants in which photosynthesis is downregulated for the induction of defense-related genes [120]. Compared to ETI, PTI is a weaker form of the immune response [31,36,63], and only involves transient induction of MAPK activation, and does not lead to photosynthesis inhibition [120]. Thus, the regulation of photosynthetic activities during defense response can be influenced by the activation kinetics of MAPK signaling depending on the nature of the immune response [120]. On the other hand, MAPK signaling mediated defense response to *Phytophthora sojae* in soybean did not involve photosynthesis inhibition [79]. These pathways were not enriched in the susceptible cultivar, suggesting that pathogen attack may have compromised these defense responses by modifying or suppressing them to reduce resistance [88]. Forty-six genes in the upregulated DEGs were associated with plant-pathogen interaction pathways. A plant-pathogen interaction pathway showed that calcium-dependent protein kinase family (CDPK), calmodulin (CaM) and calmodulin-like (CML) proteins, respiratory burst oxidase, BAK1, cysteine proteinase, serine-threonine protein kinase, Nbs-lrr resistance genes, mitogen-activated protein kinase, *Pti* (Pto interaction protein) that encodes a serine/threonine kinase, *RPM1*, as well as transcription factor WRKY, were involved in the defense response. Plant resistance to stress is accompanied by alteration in the cellular content of Ca^2+^ [148]. Ca^2+^-binding proteins, including calmodulin, calcium-dependent protein kinases detect changes in the concentration of Ca^2+^ and the signals are processed for plant defense response, biosynthesis of plant hormones, as well as plant fungal stimuli interaction [56,97,148]. These genes were upregulated in the incompatible interaction. The involvement of CaM/CML protein family in defense response has been reported [131,148]. *RPM1* confers resistance to *Pseudomonas syringae* in *A. thaliana* [81]. Plant cell death can be regulated by activity between cysteine proteases and cysteine protease inhibitor [118]. The role of Nbs-lrr domain proteins in conferring disease resistance in different plant species has been reported [138,147,151]. It can recognize effectors secreted by the pathogen and triggers ETI [149]. Nbs-lrr genes have been known to confer durable field resistance to *P. infestans* in potato [62]. About 71% of the twenty-four Nbs-lrr genes identified in the cultivar Ando were upregulated, including the gene PGSC0003DMG400007999 which was also identified among the four Nbs-lrr genes in late blight resistant potato genotype SD20 [143]. PGSC0003DMG400007999 shows high sequence similarity to disease resistance protein At4g33300-like (ADR1) [26,64,143]. Only four Nbs-lrr genes, two of which were upregulated, were identified in the susceptible cultivar Arielle. The abundance of the Nbs-lrr genes and their expression in Ando can contribute to its resistance to late blight infection. Thus, Ando could be a viable genetic resource for the exploitation of resistance genes for potato breeding. Similarly, Gu et al. [48] discovered that the resistant potato species *Solanum pinnatisectum* has more induced Nbs-lrr genes than the susceptible species *Solanum cardophyllum*.

The ROS produced by respiratory burst oxidase homologs is associated with multiple signal transduction pathways that regulate numerous biological processes in plants including disease resistance [58,140]. However, oxidative stress and disruption of cellular contents such as mitochondria can result from excessive accumulation of ROS [142,144]. This oxidative damage can be reduced by autophagy which engulfs and degrades oxidized substances, thereby removing ROS, damaged organelles, and cell structures to restore the normal growth and development of the plant [142,144]. The upregulation of autophagy-related genes observed in Ando can help to alleviate oxygen stress injury. In Arabidopsis, autophagy has been shown to play a crucial role in resistance to necrotrophic fungi pathogens [74,77].

### 4.6 Genes involved in the modification of cell wall

The cell wall forms a dynamic structure that determines the outcome of host-pathogen interactions [11]. Following pathogen invasion of plants, plant cell wall components are degraded [11]. This loss of cell wall integrity is sensed by plants and activates defense responses [11]. Genes encoding enzymes [(lipid transfer protein (LPT), glutathione-S-transferase (GST), callose synthase (CS), cinnamyl alcohol dehydrogenase (CAD)] involved in cell wall modifications during host-pathogen interactions were differentially expressed in the resistant cultivar indicating their role in host-pathogen interactions. Resistant cultivar had both upregulated and downregulated genes of GST and CAD, whereas LPT and CS were significantly downregulated indicating their expression pattern upon *P. infestans* infection. These genes were not differentially expressed in the susceptible cultivar. GSTs also play a conserved role in the detoxification of toxic compounds produced during an attack by pathogens and oxidative stress [82].

### 4.7 Regulation of genes involved in phenylpropanoid biosynthesis pathways

Lignin is an important structural component of the vascular secondary cell walls of plants. It also plays an important role in improving plant mechanical strength and vascular integrity and conferring resistance to abiotic and biotic stresses [91,133,154]. In phenylpropanoid biosynthesis, *L*-phenylalanine is converted into various aromatic compounds such as soluble phenols, flavonoids, and lignin [133]. Phenylpropanoid biosynthesis of the KEGG pathway was significantly enriched in DEGs upregulated in Arielle after infection by the late blight pathogen, indicating that increased expression of genes involved in lignin synthesis may play a positive role in responses to late blight attack. These DEGs include the 4-coumarate-CoA ligase 2 (4CL) gene, ferulic acid 5-hydroxylase, P-coumaroyl quinate/shikimate 3′-hydroxylase (C3H) gene, and peroxidase that functions in the final step of lignin biosynthesis [18]. In sugarcane, genes involved in lignin biosynthesis were upregulated in both resistant and susceptible cultivars in response to an attack by *Acidovorax avenae* [27].

## 5 Conclusions

Through transcriptomic analyses of resistant and susceptible potato cultivars infected by *P. infestans,* DEGs provided some insights into the defense mechanism activated in Ando in response to late blight infection. Analysis of DEGs, gene ontology enrichment, and KEGG pathway analysis showed that many disease-resistant pathways were significantly enriched in Ando in response to late blight infection. In response to *P. infestans* infection in Ando, a shift from photosynthesis to promoting an immune response is likely possible. Photosynthetic inhibition has also been observed in previous research on the response of potato to *P. infestans* infection [52,137,149]. The study also reveals that the SA-JA-ET pathways are also involved in defense response at 72 hpi which corresponds to the necrotrophic stage of infection in support of some other researchers [143]. This work contributes to a theoretical foundation for further study into the molecular basis underlying the interaction between highly late blight resistant cultivar Ando and *P. infestans*. However, similar to other studies that examine the defense response of potato genotype to a single *P. infestans* isolate ([54]; [33]; [143]), studies that involve transcriptional changes during potato-late blight interaction with diverse *P. infestans* isolates can be insightful [38,48], as isolates with different pathotypes can induce or repress different sets of genes that can result in a compatible or incompatible interaction [38]. Thus pathotype (involving diverse *P. infestans* isolates) by environment (different time points) interaction transcriptional changes can offer insightful information about the defense response of Ando to late blight disease. This can also mimic what is possible in the field when interaction with naturally occurring *P. infestans* populations of different pathotypes, takes place. For this experiment, we have selected one isolate that represents the most common pathotype for Europe (1.3.4.10.11). Thus, further analysis that involves also the early stages of infection: 24 hpi (early infection) and 48 hpi (biotrophy) with diverse *P. infestans* isolates should be considered to provide more insight into the interactions between *P. infestans* and potato, and the diversity of processes that regulate each pathogenicity stage, and shed light on the nature and timing of molecular responses.

## Authors' contributions

CA conceptualized the experimental design and study concept, method development, carried out the molecular analysis, submitted the data, analysed and interpreted the data, wrote the original draft of the manuscript, and critically revised the article for important intellectual content.

EK participated in the study concept and design, participated in the molecular analysis, and reviewed and edited the manuscript.

ERP and TT participated in the study concept and design, method development, reviewed and edited the manuscript.

ÜN coordinated and supervised the research, method development, conceptualized the experimental design and study concept, interpreted the data, guided the discussion of the outcomes, drafting, and revision of the article. All authors read and approved the final manuscript.

## Funding

This work was supported by grants from the 10.13039/501100008530European Regional Development Fund (Centre of Excellence EcolChange), 10.13039/501100000781European Research Council (Grant ID 8F160018PKTF), and the 10.13039/501100008099Estonian University of Life Sciences project (base funding P190259PKTT). The study used equipment purchased within the framework of the AnaEE Estonia Project (2014–2020.4.01.20-0285) and the project “Plant Biology Infrastructure-TAIM” (2014–2020.4.01.20-0282) through the EU Regional Development Fund.

## Ethics approval and consent to participate

The authors declare that all methods were carried out in accordance with relevant guidelines and regulations.

## Consent for publication

Not applicable.

## Author statement

The authors confirm that the work described in GEN-D-23-00002R2 has not been published previously and that it is not under consideration for publication in another journal while it is under consideration by the editorial board of Genomics. All named authors have agreed to its submission and have reviewed the revised version. If accepted, it will not be published elsewhere in the same form, in English, or in any other language, including electronically without the written consent of the copyright holder.

## Declaration of Competing Interest

The authors confirm that the contents of this article have no conflicts of interest.

## Data Availability

Raw data in BAM format were deposited in the National Center of Biotechnology Information (NCBI). The BioProject number is PRJNA759282 (https://www.ncbi.nlm.nih.gov/bioproject/PRJNA759282).

## Appendix A Supplementary data

Supplementary material 1 Image 1

Supplementary material 2 Image 2

Supplementary material 3 Image 3

Supplementary material 4 Image 4

Supplementary material 5 Image 5

Supplementary material 6 Image 6

Supplementary material 7 Image 7

Supplementary material 8 Image 8

Supplementary material 9 Image 9

Supplementary material 10 Image 10

Supplementary material 11 Image 11
