# Supplementary material for: Comparative transcriptome profiling of potato cultivars infected by late blight pathogen Phytophthora infestans: Diversity of quantitative and qualitative responses
Source: Genomics. 2023 Sep;115(5):110678. doi: 10.1016/j.ygeno.2023.110678 (PMC10548088; doi:10.1016/j.ygeno.2023.110678)
Supplement: Supplementary file 1 — Supplementary material 1 [file mmc1.docx]

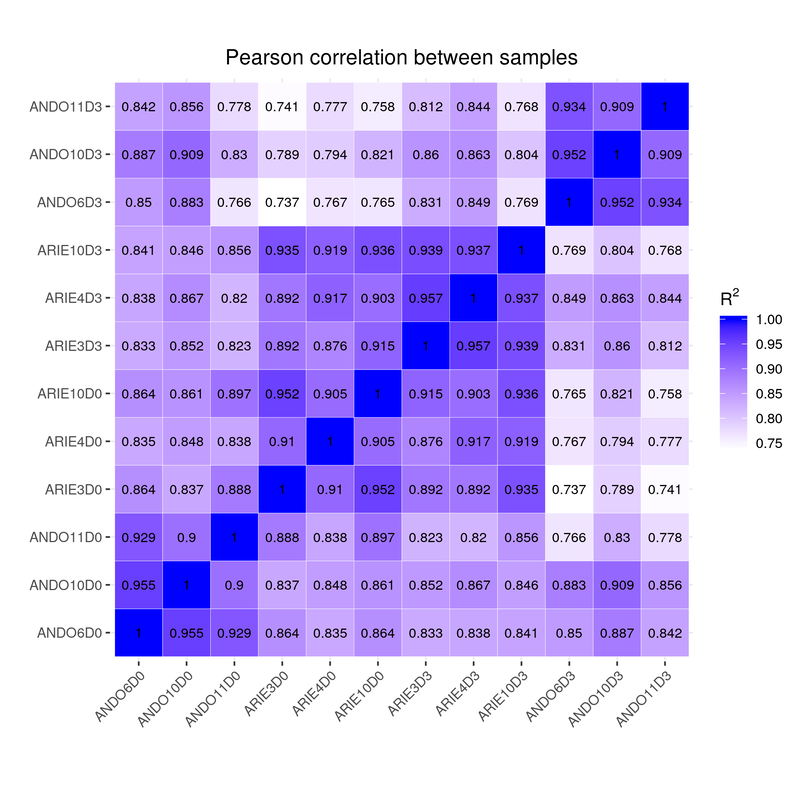

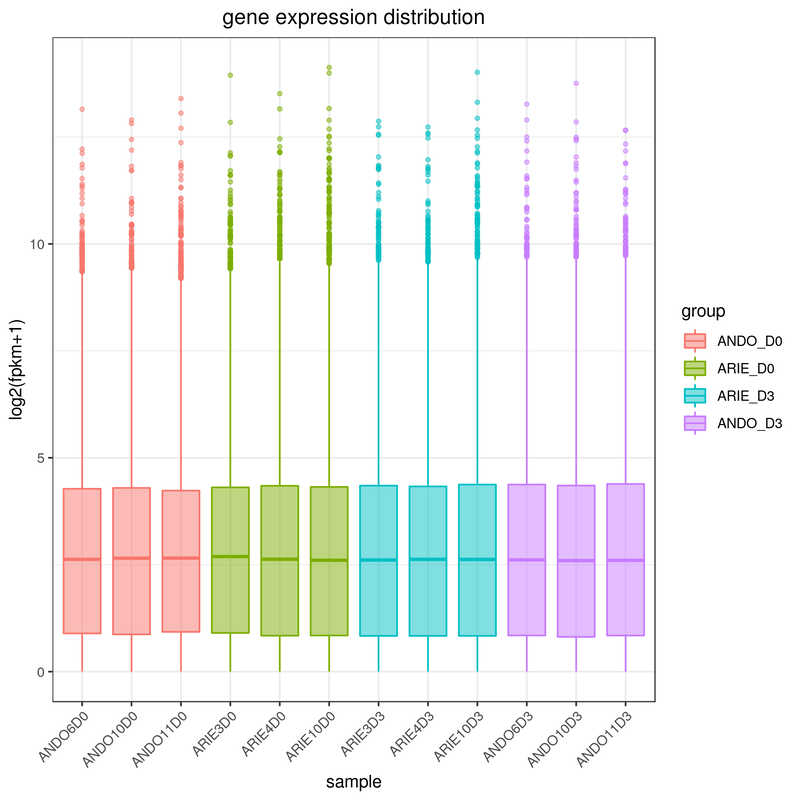


b

a

**Fig. S1**. Gene expression analysis before (D0) and after 72 hrs (D3) infection of resistant (Ando) and susceptible (Arielle) cultivars of potato leaves. **(a)** Correlation analysis between samples, where the X and Y-axis represent each sample. **(b)** Gene expression boxplot. The X-axis represents the sample name, Y-axis represents the log2FPKM value.
