# Supplementary material for: Comparative transcriptome profiling of potato cultivars infected by late blight pathogen Phytophthora infestans: Diversity of quantitative and qualitative responses
Source: Genomics. 2023 Sep;115(5):110678. doi: 10.1016/j.ygeno.2023.110678 (PMC10548088; doi:10.1016/j.ygeno.2023.110678)
Supplement: Supplementary file 2 — Supplementary material 2 [file mmc2.docx]

**Table S1. Data quality summary of sequencing of two potato (*Solanum tuberosum*) cultivars, Ando and Arielle, before and after infection by the oomycete *Phytophthora infestans***

| Sample name | Raw reads | Clean reads | Raw bases (Gb) | Clean bases (Gb) | Error rate (%) | Q20 (%) | Q30 (%) | GC (%) | Notes |
| --- | --- | --- | --- | --- | --- | --- | --- | --- | --- |
| ARIE3D0 | 51002002 | 49639930 | 15.3 | 14.9 | 0.03 | 97.33 | 92.21 | 42.53 | Replicate 1 |
| ARIE4D0 | 48686883 | 47611428 | 14.6 | 14.3 | 0.03 | 97.16 | 92.16 | 42.63 | Replicate 2 |
| ARIE10D0 | 47616981 | 46292191 | 14.3 | 13.9 | 0.03 | 97.36 | 92.22 | 43.13 | Replicate3 |
| ARIE3D3 | 52679078 | 51502614 | 15.8 | 15.5 | 0.03 | 97.23 | 92.25 | 42.37 | Replicate 1 |
| ARIE4D3 | 48763770 | 47579531 | 14.6 | 14.3 | 0.03 | 97.30 | 92.22 | 42.65 | Replicate 2 |
| ARIE10D3 | 49986534 | 48763290 | 15.0 | 14.6 | 0.03 | 97.28 | 92.43 | 42.86 | Replicate3 |
| ANDO6D0 | 54048235 | 52661995 | 16.2 | 15.8 | 0.03 | 97.16 | 92.13 | 42.51 | Replicate 1 |
| ANDO10D0 | 48396544 | 47081420 | 14.5 | 14.1 | 0.03 | 97.80 | 93.44 | 42.46 | Replicate 2 |
| ANDO11D0 | 46888162 | 45983076 | 14.1 | 13.8 | 0.03 | 97.13 | 92.06 | 42.7 | Replicate3 |
| ANDO6D3 | 47679810 | 46593040 | 14.3 | 14.0 | 0.03 | 97.35 | 92.25 | 42.26 | Replicate 1 |
| ANDO10D3 | 48864894 | 47687338 | 14.7 | 14.3 | 0.03 | 97.27 | 92.35 | 42.38 | Replicate 2 |
| ANDO11D3 | 47991170 | 46900644 | 14.4 | 14.1 | 0.03 | 97.05 | 91.86 | 42.38 | Replicate3 |
| Total | 592604063 | 578296497 | 177.8 | 173.6 | 0.03% | 97.29% | 92.30% | 42.57% |  |

Q20 (%) and Q30 (%) are the percentages of reads with Phred quality scores >20 and >30, respectively.

GC content (%) is G + C bases as a percentage of total bases.

Error rate = 0.03% in all samples; error rate (%) is base error rate of whole sequencing.

ARIE is cultivar Arielle; ANDO is cultivar Ando; D0 and D3 are before and 72 hour post-inoculation, respectively.
