# Supplementary material for: Comparative transcriptome profiling of potato cultivars infected by late blight pathogen Phytophthora infestans: Diversity of quantitative and qualitative responses
Source: Genomics. 2023 Sep;115(5):110678. doi: 10.1016/j.ygeno.2023.110678 (PMC10548088; doi:10.1016/j.ygeno.2023.110678)
Supplement: Supplementary file 3 — Supplementary material 3 [file mmc3.docx]

**Table S2. Total reads of *S. tuberosum* cv. Ando and cv. Arielle mapped to the reference genome of *S. tuberosum***

| Sample name | ANDO10D0 | ANDO10D3 | ANDO11D0 | ANDO11D3 | ANDO6D0 | ANDO6D3 | ARIE10D0 | ARIE10D3 | ARIE3D0 | ARIE3D3 | ARIE4D0 | ARIE4D3 |
| --- | --- | --- | --- | --- | --- | --- | --- | --- | --- | --- | --- | --- |
| Total reads | 94162840 | 95374676 | 91966152 | 93801288 | 105323990 | 93186080 | 92584382 | 97526580 | 99279860 | 103005228 | 95222856 | 95159062 |
| Total mapped reads | 72194768 | 69916858 | 81144729 | 81659751 | 76233364 | 65347591 | 82616931 | 85957110 | 87602157 | 89984280 | 83230233 | 83798563 |
| Uniquely mapped reads | 69565910 | 67708519 | 78272702 | 79062028 | 73648381 | 63242157 | 79506126 | 82804669 | 84506508 | 87141797 | 80520746 | 81183237 |
| Multiple mapped reads | 2628858 | 2208339 | 2872027 | 2597723 | 2584983 | 2105434 | 3110805 | 3152441 | 3095649 | 2842483 | 2709487 | 2615326 |
| Total mapping percentage | 76.67% | 73.31% | 88.23% | 87.06% | 72.38% | 70.13% | 89.23% | 88.14% | 88.24% | 87.36% | 87.41% | 88.06% |
| Percentage of the uniquely mapped read | 73.88% | 70.99% | 85.11% | 84.29% | 69.93% | 67.87% | 85.87% | 84.90% | 85.12% | 84.60% | 84.56% | 85.31% |
| Multiple mapping percentage | 2.79% | 2.32% | 3.12% | 2.77% | 2.45% | 2.26% | 3.36% | 3.23% | 3.12% | 2.76% | 2.85% | 2.75% |

Sample codes as in Table S1
