# Supplementary material for: Comparative transcriptome profiling of potato cultivars infected by late blight pathogen Phytophthora infestans: Diversity of quantitative and qualitative responses
Source: Genomics. 2023 Sep;115(5):110678. doi: 10.1016/j.ygeno.2023.110678 (PMC10548088; doi:10.1016/j.ygeno.2023.110678)
Supplement: Supplementary file 5 — Supplementary material 5 [file mmc5.docx]

**Table S4. Differentially expressed genes in resistant (Ando) and susceptible (Arielle) cultivars.**

| **Cultivar** | **Up- or down-regulated** | **No. of all regulated DEGs** | **No. of top 5% of regulated DEGs** | **Range of Log_2_FC of the top 5% of DEGs** | **Mean Log_2_FC of the top 5% of DEGs** |
| --- | --- | --- | --- | --- | --- |
| Ando | Up | 2893 | 145 | 3.98∼10.09 | 5.20±0.09 |
| Arielle | Up | 658 | 33 | 3.62∼6.67 | 4.66±0.13 |
| Ando | Down | 3034 | 152 | -3.52 ∼ -9.58 | -4.62±0.09 |
| Arielle | Down | 503 | 25 | - 2.93∼ -7.93 | -4.02±0.24 |
| **Cultivar** | **Up- or down-regulated** | **No. of all specifically regulated DEGs** | **No. of top 5% of specifically regulated DEGs** | **Range of Log_2_FC of the top 5% of DEGs** | **Mean Log_2_FC of the top 5% of DEGs** |
| Ando | Up | 2592 | 130 | 3.95∼10.09 | 5.18±0.10 |
| Arielle | Up | 357 | 18 | 4.22∼6.67 | 4.98±0.17 |
| Ando | Down | 2833 | 142 | -3.42∼-9.58 | -4.58±0.10 |
| Arielle | Down | 302 | 15 | -3.15∼-6.49 | -4.09±0.25 |
